# Set-shifting, central coherence and decision-making in individuals recovered from anorexia nervosa: a systematic review

**DOI:** 10.1186/s40337-019-0251-5

**Published:** 2019-06-20

**Authors:** Tone Seim Fuglset

**Affiliations:** Division of Mental Health and Addiction, Møre and Romsdal Hospital Trust, Parkvegen 84, 6412 Molde, Norway

**Keywords:** Anorexia nervosa, Recovered, Executive functions, Systematic review

## Abstract

**Background:**

The aim of this study was to review the existing literature and evaluate whether deficits in set-shifting, central coherence and decision-making persist in individuals recovered from anorexia nervosa (AN-REC).

**Method:**

A systematic review approach was used. Literature was identified via searches in PubMed, PsychInfo and Embase database. The main search resulted in 158 articles. After exclusion of 135 articles, 23 articles were included in the review.

**Results:**

The majority of studies on set-shifting showed that set-shifting difficulties persist after recovery. Central coherence might also be trait related, however findings are inconsistent. Few studies have investigated decision-making in AN-REC, however those studies that do exist suggest that decision-making is not impaired in AN-REC.

**Conclusions:**

Novel treatment strategies based on neuroscience research are emerging, focusing on targeting the underlying mechanisms of the illness, including neuropsychological functioning. Whether these functions are trait or state related could have implications for how they are targeted in treatment.

## Plain English summary

Studies have consistently shown that patients with anorexia nervosa have altered neuropsychological functions in some cognitive domains. An important question is whether these difficulties persist after the patients have recovered from their illness. If they *do* persist, this could be related to traits and might be considered as predisposing factors of the illness. If these difficulties normalize with recovery, they are likely a consequence of the illness state. In this study, relevant scientific literature was gathered to investigate whether deficits in the neuropsychological functions i) set-shifting, ii) global processing and iii) decision-making still persists after recovery. In total, 23 studies were included in this review article. The majority of studies on set-shifting indicate that this function is impaired in individuals recovered from anorexia nervosa. Studies including global processing and decision-making are more unclear, as the results are inconsistent.

As anorexia nervosa is an illness that is difficult to treat, there is an urgent need for new and better treatment methods. There has been an increased interest in incorporating findings from neuroscientific research in developing new treatment strategies. These strategies are focusing on the underlying mechanisms of the illness, such as neuropsychological functioning. It would be beneficial to know whether neuropsychological deficits are related to predisposing traits or is a consequence of the illness state, as it might influence how we clinically approach the symptoms and behaviors that we see in anorexia nervosa.

## Background

Anorexia nervosa (AN) is considered to be one of the most difficult psychiatric disorders to treat. There is a lack of efficient treatment approaches, especially for adult patients. The aetiology of AN is still unclear, however neuroscientific research has contributed to the development of theoretical models of the illness, linking the core symptoms of AN such as restrictive eating to alterations in brain circuits [1–4].

It has been suggested that neuropsychological deficits mediate between underlying neurobiological functioning and the symptom and behaviors that we see in these patients [5]. Neuropsychological studies have repeatedly demonstrated significant deficits in executive functions such as set-shifting, central coherence and decision-making. Set-shifting refers to the ability to move back and forward between different tasks or mental sets, and is often used as a measure of cognitive flexibility. Patients with AN often have a rigid thinking style, which also involves eating and weight. These patients also often struggle with adapting to environmental changes. Studies have consistently shown that AN patients perform worse on set-shifting tasks compared to healthy controls [6]. Central coherence refers to a bias towards processing details (local processing) at the expense of paying attention to the bigger picture (global processing). A range of studies have demonstrated weak central coherence in patients with AN, suggesting that these patients have a local processing style, and show greater global integration difficulties [7]. Furthermore, decision-making is also likely impaired in patients with AN, as demonstrated repeatedly by several studies (rewieved in [8]). Clinical observations of patients often show that they struggle with making and trusting decisions. This could apply to decisions regarding food and what to eat, but also in other settings outside a meal situation.

A commonly raised question in this field is whether neuropsychological alterations such as deficits in set-shifting, central coherence and decision-making persist after recovery. If they *do* persist, they could be stable traits and not related to the state of the illness. Considering an increased interest in neuroscientific-based treatment methods that has emerged during recent years, it would be beneficial to determine whether such neuropsychological deficits are trait – or state related as it could have implications for how we approach these deficits clinically.

### Aim of study

The aim of the present study is to systematically review the literature to evaluate whether deficits in set-shifting, central coherence and decision-making persist in individuals recovered from AN (AN-REC), and to discuss whether these neuropsychological functions are likely to be state or trait related.

## Methods

### Search strategy and selection of studies

Relevant literature was identified via searches in PubMed, PSychINFO and Embase databases using the search terms [anorexia nervosa] AND [recovered OR recovery OR mental health recovery] AND [set shifting OR central coherence OR decision making].

**Fig. 1:**
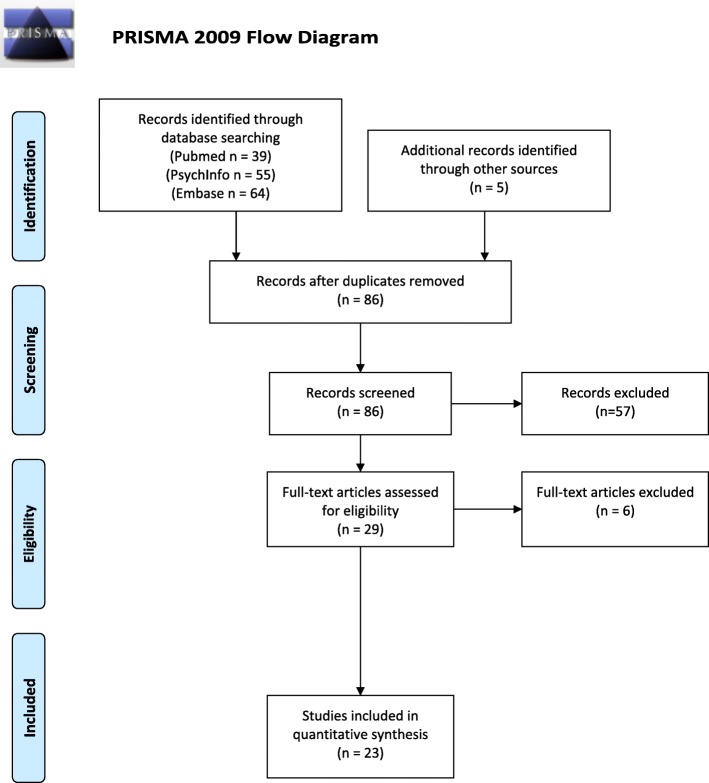
PRISMA 2009 Flow Diagram

In total, the main search resulted in 158 articles. A supplemental search was performed by a manual search in Google Scholar, which resulted in five studies. After removal of duplicates, a total of 86 articles were assessed for eligibility. Studies were included if they met the following inclusion criteria:

1. Study included an AN-REC or an AN weight recovered (AN-WR) sample and comparison with a healthy control group/normative scores.
2. Manuscript written in English.
3. Study published in peer-reviewed journals.
4. Abstracts and titles were screened for relevance and eligibility, and 57 articles were excluded. The full texts of the remaining 29 articles were examined in more detail. Of these, six studies were excluded as they were not relevant for the current review (did not include recovered patients or did not include relevant neuropsychological tests). In all, 23 articles were included in the present study. Figure 1 illustrates a PRISMA flow chart of the search strategy.

### Study characteristics

One study included both males and females in their sample [9], another study did not specify the gender of the participants [10], and the remaining studies included females only. Nineteen studies included an adult sample, (age ≥ 18 years), whereas only two studies included an adolescent sample (age < 18 years) [11, 12]. Two studies included both adolescents and adults [13, 14]. Definition of recovery varied across studies, see Table 1 for an overview of the different definitions of recovery.

**Table 1:** Overview of criteria for recovery and duration of recovery. The articles are presented chronologically according to year of publication

Authors	Criteria	Duration of recovery
Tchanturia et al., 2002 [10]	Criteria for AN in the past	Minimum 1 year
	BMI 19–24	
	Regular menstruation	
	Normal eating patterns	
Tchanturia et al., 2004 [15]	Stable BMI	Minimum 1 year
	Regular periods	
	No psychotropic medication	
Holliday et al., 2005 [16]	Normal weight	At least 1 year
	Regular menses	
Tchanturia et al., 2007 [17]	BMI between 20 and 25	At least 1 year
	Regular menstruation	
Nakazato et al., 2009 [18]	History of AN diagnosis according to the DSM-IV	At least 1 year
	BMI between 18.5–24	
	Regular menstrual cycles	
	Binge and purge behavior absent	
	No prescribed psychotropic medication	
Lopez et al., 2009 [19]	BMI between 19 and 26	In the current year
	No binging, purging, food restriction or excessive exercise	
Nakazato et al., 2010 [20]	History of AN diagnosis according to the DSM-IV	At least 1 year
	BMI between 18.5–24	
	Regular menstrual cycles	
	Binge and purge behavior absent	
	No prescribed psychotropic medication	
Roberts et al., 2010 [21]	Healthy BMI (> 17.5)	1 year
	Regular periods	
	No AN or BN behaviors	
Tenconi et al., 2010 [14]	Normal weight	At least 3 years
	Regular menses	
	No ED symptoms and good social and interpersonal outcome	
Harrison et al., 2011 [13]	Restored regular menstruation	At least 1 year
	No scores above 4 on EDE-Q	
	BMI > 18.5	
Bühren et al., 2012 [12]	Patients were tested before and after weight rehabilitation	Mean duration of hospital treatment was 122 +/− 33 days, range: 57–193 days
Danner et al., 2012 [22]	BMI > 18.5 and recovered menstrual cycle	At least 12 consecutive months
	EDE-Q and BDI not different from HC	
Favaro et al., 2012 [23]	Asymptomatic	At least 3 months
Harrison et al., 2012 [24]	Restored regular menstruation	At least 1 year
	No scores above 4 on EDE-Q	
	BMI > 18.5	
Lindner et al., 2012 [25]	No DSM-IV criteria	At least 1 year
	BMI between 18.5 and 26	
	Regular menstrual cycles	
	No ED specific cognitions	
Tchanturia et al., 2012 [26]	BMI > 18.5	At least 1 year
	Restored menstruation	
	Absence of ED behaviors	
Lindner et al., 2013 [27]	No DSM-IV criteria	At least 1 year
	BMI between 18.5 and 26	
	Regular menstrual cycles	
	No ED specific cognitions	
Lindner et al., 2014 [28]	No DSM-IV criteria	At least 1 year
	BMI between 18.5 and 26	
	Regular menstrual cycles	
	No ED specific cognitions	
Ritschel et al., 2015 [29]	If > 18 years old, BMI > 18.5	At least 6 months
	If < 18 years old, BMI > 10th BMI percentile	
	Menstruation	
	No binge, purge or restrictive eating pattern	
Talbot et al., 2015 [9]	BMI ≥ 18.5	Past 3 months (at minimum)
	No binging, purging, restricting and driven or compulsive exercise	
	Scores on all subscales of EDE-Q within 1 SD of population	
	norms	
Ely et al., 2016 [30]	Stable weight between 90 and 120% of ideal body weight	Prior 12 months
	Regular menstrual cycles	
Sultson et al., 2016 [31]	BMI > 18.5	At least 12 months
	Recovered menstrual cycle	
	No differences from HC on the EDE-Q	
Bentz et al., 2017 [11]	If > 16 years old, BMI > 18.5	At least 1 year
	If 14–15 years old, BMI-percentile corrected for age > 25th percentile	
	No present ED pathology	
	Global EDE-Q score within 1 SD of non-AN mean	
	MROAS ≥9	

*AN* anorexia nervosa, *BDI* Beck’s depression inventory, *BMI* body mass index, *BN* bulimia nervosa; *DSM-IV* Diagnostic and Statistical Manual of Mental Disorders, 4th Edition, *ED* eating disorders, *EDE-Q* Eating disorders examination questionnaire *HC* healthy controls, *MROAS* Morgan Russel Outcome Assessment Schedule, *SD* standard deviation

## Results

The studies included in this review were published between 2002 and 2017. The distribution across neuropsychological functions were as follows:

1. Set-shifting: 16
2. Central coherence: 8
3. Decision-making: 4

### Set-shifting

A variety of tests was used to investigate set-shifting, including Fixed Set Task, Cognitive Shift Task, Trail Making Task (TMT), Brixton Test, CatBat Test, Uznadze Illusion Task, Verbal Fluency Test, Haptic Illusion Test, Wisconsin Card Sorting Test (WCST) and Berg’s Card Sorting Test. The majority of studies found poorer set-shifting in AN-REC compared to healthy controls [9, 10, 14–16, 22, 24, 26, 28]. They demonstrate that set-shifting difficulties persist after recovery, and that AN-REC does not differ from acute AN patients. Results from two of the studies showed a trend towards poorer set-shifting, but the findings were not significant [30, 31]. Similarly, one study found some evidence for poorer set-shifting in AN-REC [21]. Four studies reported no differences between AN-REC and healthy controls, suggesting that there are no set-shifting difficulties in recovered patients [11, 12, 18, 20].

### Central coherence

Most studies used the Rey Complex Figure Test (RCFT) to investigate central coherence, but also the Embedded Figures Test, Sentence Completion Task, Homograph Reading Task, National Adult Reading Test and Fragmented Pictures Task were utilized. Four of eight studies found no differences between the AN-REC and the acute group, suggesting weak central coherence in the AN-REC group [13, 14, 19, 24]. One study found that AN-REC had no inefficiencies in global processing but a superior local processing [27]. Three studies found no significant differences in central coherence between the AN-REC group and healthy controls [9, 11, 22].

### Decision-making

Only four studies have investigated decision-making in AN-REC. In a study using intertemporal choice task, no differences were observed between AN-REC and healthy controls (HC) [29]. Similar results were found in a study using the Iowa gambling task (IGT) [17]. Another study using the IGT actually reported better performance in the AN-REC group [25]. On the other hand, the same test resulted in poorer decision-making in AN-REC Table 2 [22].

**Table 2:** Review of the literature of set-shifting, central coherence and decision-making in individuals recovered from anorexia nervosa. The articles are presented chronologically according to year of publication

Authors	Neuropsychological function	Tests	Aim of study	Sample	Age Mean (SD)	Main findings	Effect size (test)	Conclusion
Tchanturia et al., 2002 [10]	Set-shifting	Fixed set task	To examine perceptual and cognitive set-shifting	AN = 30	AN = 25.2 (6.7)	Significant differences between AN/AN-REC compared to HC	*d* = 1.37 (Fixed set task)	Impaired set-shifting could represent a vulnerability factor for AN
		Cognitive shift task		AN-REC = 16	AN-REC = 30.0 (6.0)			
				HC = 23			*d* = 0.72 (Cognitive shift task, perseverations)	
					HC = 27.6 (6.4)			
Tchanturia et al., 2004 [15]	Set-shifting	TMT	To investigate set-shifting in current and past AN patients	AN = 34	AN = 27.2 (8.3)	Set-shifting difficulties was observed in the AN group, and to a lesser degree in the AN-REC group	*d* = 0.87 (Picture set test)	Some aspects of set-shifting in AN appear to be a trait rather than state marker
		Brixton test		AN-REC = 18	AN-REC = 28.4 (6.8)			
		Picture set test		HC = 36			*d* = 0.88 (illusions)	
		Cat bat tests			HC = 25.9 (4.8)			
		Uznadze illusion task						
Holliday et al., 2005 [16]	Set-shifting	Haptic illusion task	To investigate whether set-shifting difficulties are familial	47 pairs of sisters disconcordant for AN: AN-REC = 23	AN (AN-REC + AN+ AN-WR) = 26.3 (10.2)	Set-shifting difficulties persists after recovery	N/A	Suggest that set-shifting difficulties are trait characteristics and may inform the search for the endophenotype of AN
		Brixton Test						
		TMT						
		CatBat task			HS = 27.6 (9.6)			
				AN = 21				
				AN-WR = 3	HC = 26.5 (6.1)			
				HS = 47				
				HC = 47				
Tchanturia et al., 2007 [17]	Decision making	IGT	To determine whether decision-making is impaired in AN-REC	AN = 29	AN = 28.5 (9.17)	No differences between AN-REC and HC. AN grop did poorer than AN-REC and HC	–	Impaired IGT performance could be a consequence of starvation
				HC = 29	HC = 26.3 (7.9)			
				AN-REC = 14	AN-REC = 28.9 (7.4)			
Lopez et al., 2009 [19]	Central coherence	Embedded figures test Unsegmented/segmented block design	To examine whether AN-REC women have weak central coherence	AN-REC = 42	AN-REC = 25	Weak central coherence in the AN-REC group	*d* = 0.53 (Rey order)	Suggest that weak central coherence is a stable trait and an endophenotype for AN
				HC = 42	HC = 26			
							*d* = 0.73 (Style)	
		RCFT					*d* = 0.57 (CC Index)	
		SCT						
							*d* = 1.15 (SCT)	
		Homograph reading task						
Nakazato et al., 2009 [18]	Set-shifting	WCST	To establish whether set-shifting difficulties are present in AN and AN-REC	AN = 29	AN = 28.3 (11.0)	No significant differences between AN-REC and HC	–	Strongly suggest that impairment in set-shifting normalizes with recovery
				AN-REC = 18	AN-REC = 32.2 (11.1)			
				HC = 28				
					HC = 26.9 (5.8)			
Nakazato et al., 2010 [20]	Set-shifting	WCST TMT	Determine whether serum glutamine is associated with set-shifting ability	AN = 27	AN = 27.7 (10.6)	No differences between AN-REC and HC	–	Serum concentrations does not appear to be associated with executive functions
					AN-REC = 32.2 (11.1)			
					HC = 26.9 (5.8)			
				AN-REC = 18				
				HC = 28				
Roberts et al., 2010 [21]	Set-shifting	TMT	To add clarity to set-shifting in EDs	AN-R = 35	AN-R = 23.71 (6.39)	Some evidence for poorer set shifting in AN – REC	*d* = 0.64 (WCST)	It is likely a familial trait, and related to the maintenance of the illness
		WCST		AN-BP = 33				
		Brixton test		BN = 30	AN-BP = 25.58 (7.64)			
		Haptic illusion		AN-REC = 30				
					BN = 26.43 (6.84)			
				AN sister = 30				
				BN sister =20	AN-REC = 32.13 (11.64)			
				HC = 88				
					AN sister = 24.23 (6.44)			
					BN sister = 27.60 (8.71)			
					HC = 28.43 (8.47)			
Tenconi et al., 2010 [14]	Set-shifting Central coherence	WCST	To explore suitability for endophenotypes in ED	AN = 60	AN = 26.2 (6.9)	No differences between AN and AN-REC	–	Impaired set-shifting and low central coherence might be an endophenotype of AN
		TMT		AN-WR = 63	HC = 27.4 (4.5)			
		RCFT		AN-REC = 30				
				HC = 120				
Harrison et al., 2011 [13]	Central coherence	RCFT GEFT FPT	To replicate findings of weak central coherence	AN = 50	ED group = 27.13 (9.3)	Superior detail processing skills was associated with having an ED and recovered from AN	*d* = 0.58 (RCFT)	AN-REC are skilled at detailed processing. Weak central coherence may be a factor that perpetuates ED behaviors
							*d* = 0.31 (GEFT)	
				BN = 48				
				AN-REC = 35	AN-REC = 29.00 (10.62)			
				HC = 89				
					HC = 28.5 (9.93)			
Bühren et al., 2012 [12]	Set-shifting	Visual set-shifting task	To investigate set-shifting in AN before and after weight gain	AN = 28	AN = 15.6 (1.5)	No deficits in set-shifting abilities.	–	Speculate that findings could be related to short duration of illness
				HC = 27	HC = 15.0 (1.7)			
Danner et al., 2012 [22]	Set-shifting Central coherence Decision making	BCST	To examine set-shifting in women AN-REC	AN = 16	AN = 25.63 (5.41)	Poor set-shifting and decision making in AN-REC compared to HC. No differences between the groups in central coherence	*d =* 1.16 (BCST perseverative errors)	Suggest that impaired decision-making and set-shifting is a stable trait in AN
		RCFT		AN-REC = 15				
		IGT		HC = 15	AN-REC = 24.33 (4.72)			
							*d* = 1.14 (BCST total errors	
					HC = 25.8 (4.69)			
							*d* = 1.05 (BCST categories completed)	
							*d* = 1.36 (Total IGT score)	
Favaro et al., 2012	Central coherence	RCFT	To explore functional connectivity of networks involved in visuospatial and somatosensory processing	AN = 29	AN = 25.8 (6.9)	No differences between AN-REC and HC in central coherence	–	Results could be due to a milder form of the illness in the AN-REC group, measures by minimum BMI and duration of illness
				AN-REC = 16	AN-REC = 23.8 (4.8)			
				HC = 26				
					HC = 26.7 (6.7)			
Harrison et al., 2012 [24]	Set-shifting	WCST	To explore cognitive and social emotional functioning	ED = 100 ED-REC = 35 HC = 90	N/A	ED-REC did not differ from the acute group.	–	Suggest that cognitive style is a trait.
	Central coherence	Brixton task FPT						
		RCFT						
Lindner et al., 2012 [25]	Decision making	IGT	To examine decision-making and planning in AN-REC	AN-REC = 100	AN-REC = 34.49 (7.13)	AN-REC did better in decision-making	*d* = 0.35 (IGT)	Findings are in contrast to previous findings of impaired decision making in AN-REC
				HC = 100				
					HC = 34.53 (7.26)			
Tchanturia et al., 2012 [26]	Set-shifting	WCST	To explore WCST performance and other clinical outcomes	AN = 171	AN = 25.4 (8.2)	AN-REC showed better performance than AN, but did more perseverative errors than HC	*d* = 0.50 (WCST perseverative errors)	This large dataset supports previous studies which indicate poor cognitive flexibility in people with EDs.
				BN = 82	BN = 27.3 (8.3)			
				AN-REC = 90	AN-REC = 30.7 (11.1)			
				HC = 199				
					HC = 27.7 (8.8)			
Lindner et al., 2013 [27]	Central coherence	RCFT	To examine central coherence as a possible endophenotype in AN	AN-REC = 100	AN-REC = 34.49 (7.13)	AN-REC group showed better accuracy in the copy condition	*d* = 0.46 (RCFT copy condition)	No inefficiencies in global processing but a superior local processing
				HC = 100				
					HC = 34.53 (7.26)			
Lindner et al., 2014 [28]	Set-shifting	BCST	To explore whether set-shifting is inefficient after full recovery of AN	AN-REC = 100	AN-REC = 34.49 (7.13)	AN-REC achieved fewer categories, more perseverations and spent less time for shifting set	*d* = 0.39 (BCST categories)	Suggest that set-shifting is inefficient after full recovery
				HC = 100				
					HC = 34.53 (7.26)		*d* = 0.35 (BCST perseverations)	
							*d* = 0.36 (BCST reaction time)	
Ritschel et al., 2015 [29]	Decision making	Intertemporal choice task	To investigate delay discounting in ill and recovered AN	AN = 34	AN = 15.29 (2.7)	No group differences in delay discounting	–	Suggest that delay discounting is not a trait marker for AN
				AN-REC = 33	AN-REC = 21.67 (3.1)			
				HC = 54				
					HC = 18.75 (4.4)			
Talbot et al., 2015 [9]	Set-shifting and central coherence	WCST Matching familiar figures test RCFT	To investigate whether impaired set-shifting and weak central coherence represent state or trait	AN = 24	AN = 21.0 (18-	Poorer set-	*d* = 0.79 (WCST)	This study found no
				AN-WR = 10	27)^a^	shifting in AN –REC and AN-WR compared to HC.		support that weak central coherence is an endophenotype for AN
				AN-REC = 15	AN-WR = 21.5 (19–25) ^a^			
				HC = 43				
					AN-REC = 24.0 (21–32) ^a^	No differences between the groups on measures of local and global processing		
					HC = 19.0 (18–25) ^a^			
Ely et al., 2016 [30]	Set shifting	Color-Word interference test	To identify deficits specific to inhibition or task-switching	AN-REC = 47	AN-REC = 26.68 (1.83)	Differences between the groups trended toward – but was not – significant	–	Suggest that cognitive control impairments in AN is related to anxiety, and not a neuropsychological deficit
				HC = 24				
					HC = 25.08 (6.11)			
Sultson et al., 2016 [31]	Set-shifting	BCST	To investigate whether activation in frontal and parietal regions is associated with set-shifting ability	AN = 16	AN = 25.57 (5.8)	No differences between AN-REC and HC, but a trend towards more perseverative errors and completing less categories than HC	–	Higher activation in frontal regions involved in self-referential processing and cognitive control is associated with poor set-shifting ability in AN-REC
				AN-REC = 15	AN-REC = 24.79 (4.5)			
				HC = 15				
					HC = 25.80 (4.7)			
Bentz et al., 2017 [11]	Set shifting and local processing	TMT	To investigate impairments of social functioning and potential associations with neurocognitive functions	AN = 43	AN = 16.1 (1.5)	No differences between the groups on neurocognitive functions	–	Young AN and AN-REC did not differ from HC
		GEFT		AN-REC = 28	AN-REC = 18.4 (1.6)			
				HC = 41				
					HC = 17.7 (2.2)			

Effect sizes for significant differences between AN-REC and HC. Cohen’s *d* effect size: small (*d* = 0.2), medium (*d* = 0.5) and large (*d* = 0.8)

*AN* anorexia nervosa, *AN – REC* anorexia nervosa recovered, *AN – WR* anorexia nervosa weight restored, *BN* bulimia nervosa, *BCST* Berg’s Card Sorting Test, *ED’s* eating disorders, *CC index* Central Coherence index, *ED-REC* eating disorders recovered, *FPT* Fragmented pictures task, *GEFT* Group Embedded Figures Test, *HC* healthy controls, *HS* Healthy sister, *IGT* Iowa Gambling Task, *N/A* Not applicable, *RCFT* Rey Complex Figure Test, *SCT* Sentence completion task, *TMT* Trail making test, *WCST* Wisconsin Card Sorting Test. The main findings represents significant differences in the experimental group (AN-REC) compared to a healthy control group

^a^ Median values with upper and lower quartiles

## Discussion

The aim of this review was to systematically examine the literature to evaluate whether deficits in set-shifting, central coherence and decision-making persist in individuals recovered from AN-REC, and to discuss whether these neuropsychological functions are likely to be state or trait related. The current review used 23 studies. The majority of studies in set-shifting suggest that this function is impaired in AN-REC. The evidence base for central coherence, on the other hand, is more unclear as findings are inconsistent. Few studies have investigated decision-making in AN-REC, however most studies so far suggest that this function is not different from healthy controls.

### State or trait?

The majority of findings in this review suggest that set-shifting difficulties could be trait characteristics rather than a state marker. Tchanturia et al. suggest that impaired set-shifting could represent a vulnerability factor for AN [10] and that some aspects of set-shifting could be a trait rather than a state marker [15]. Likewise, in the study by Holliday et al. [16] there were no differences between acute AN and those who had fully recovered from AN. A common question is whether neuropsychological deficits in AN-REC represent scarring effects after many years of starvation and underweight. Due to methodological challenges, this is difficult to determine, and still debated. However, Holliday et al. [16] further suggest that reduced cognitive and perceptual flexibility could reflect a familial trait that is associated with a greater risk of developing AN, and not a scar due to the illness. Set-shifting difficulties as a trait is also supported by Harrison et al. [13] who reported a less adaptive cognitive style and social and emotional profile would be a trait that is associated with the eating disorder. They also suggest that this could be a maintaining factor, having the least adaptive cognitive style would be associated with a more chronic and severe form of the illness. Furthermore, they found that both individuals currently ill and recovered from AN have a fragmented perseverative cognitive style with social and emotional difficulties. A rigid cognitive style accompanied with social emotional difficulties was associated with a more persistent and severe form of illness. Danner et al. [22] also suggest that this is a stable impairment, as their results show that problems with set-shifting persist in AN-REC. Similar results was reported in the study by Talbot et al. [9], who found that individuals that have fully recovered from AN, still have impairments in set-shifting. They claim that full recovery from AN does not signify normal neuropsychological functioning. The results from Lindner et al. [28] showed that AN-REC showed inefficient set-shifting or flexibility, i.e. they stayed longer to the old rule instead of changing to the new rule. In summary, these studies suggest that difficulties with set-shifting could be trait-related.

On the other hand, some studies failed to detect any differences between AN-REC and HC on a set-shifting task. Bentz et al. [11] did not find any differences between the groups (AN, AN-REC and HC) in cognitive flexibility. Similarly, Bühren et al. [12] and Nakazato et al. [18] found no deficits in set-shifting in AN-REC. Ely et al. [30] failed to show any differences in cognitive inhibition and set-shifting in a remitted sample of AN patients and HC. They suggests that cognitive control impairments in AN is related to anxiety and does not reflect a neuropsychological deficit.

Results from studies on central coherence are more ambiguous. Based on the existing literature, it is difficult to determine whether deficits in central coherence is state or trait-related. Findings from some studies in this review show that AN-REC have persistent deficits in central coherence, while other studies indicate normal global processing in AN-REC. In the study by Danner et al. [22], those with poor set-shifting also showed weaknesses in central coherence. They suggest that these deficits could be specific to a subpopulation of individuals with AN, and that this could be linked to a rigid thinking style. Lopez et al. [19] found that AN-REC did “extremely well” on the EFT, which benefit from enhanced detailed function and suggest that weak central coherence could be a stable trait. Likewise, Harrison et al. [13] reported that AN-REC are skilled at detailed processing and that weak central coherence may be a factor that perpetuates eating disorder behavior. They also suggest that cognitive style could be a trait of the illness [24].

Lindner et al. [27] did not find any inefficiency in global processing in the recovered group. Similarly, Talbot et al. [9] did not find any differences in measures of local and global processing. Therefore, based on the existing literature, caution should be made when determining whether central coherence is related to state or trait of AN.

Decision-making is a complex construct, and few studies have investigated decision-making in AN-REC. However, according to the existing studies, it may seem like AN-REC does not have difficulties with decision-making tasks. In a study using the IGT, Tchanturia et al. [17] found that the AN-REC group performed just as well as the HC group, and they question whether poor decision-making is related to starvation. Another study using the IGT actually reported that AN-REC did better than HC [25]. One possible explanation could be that the AN-REC group have lower risk-taking behaviors. In a delay discounting task which included a monetary reward, Ritschel et al. [29] found no differences in delay discounting between AN-REC and HC. They suggest that altered self-control might be limited to disorder-relevant reinforcers, such as food. One study, using the IGT, reported that decision-making was impaired in AN-REC, and suggest that impaired decision-making is a stable trait in AN [22].

### Neuropsychological endophenotypes in AN?

There has been an increased interest in determining possible endophenotypes for eating disorders. Endophenotypes can be defined as observable behavioral characteristics that underlie and contribute to disease vulnerabilities, but are not a part of the disease itself. There are three criteria of an endophenotype: i) the candidate trait is associated with the illness, ii) is state-independent, and iii) is present in unaffected family members [32]. The endophenotypes for AN are still unclear, however it has been argued that both set-shifting and central coherence could be potential endophenotypes for AN [9].

In the current review, persistent impairments in set-shifting is the most robust finding, as opposed to central coherence and decision-making. As an endophenotype needs to be trait-related and also present in family members, it is worth mentioning that two studies in this review also included unaffected family members. Holliday et al. [16] found that set-shifting deficits in women with AN were shared with their healthy sisters. Both patients and their sisters took longer to set-shift on the CatBat cognitive set-shifting task and the Haptic Illusion task. They suggest that their study provides further support for the possibility that set-shifting may be a part of the endophenotype in AN. Similarly, Tenconi et al. [14] found that AN patients and their sisters share impairments in set-shifting. They also suggest that set-shifting is a cognitive endophenotype for AN. Poor set-shifting as an endophenotype could contribute to rigid and compulsive behaviors that are often seen in individuals with AN.

Further research with unaffected family members and AN-REC is needed to further investigate endophenotypes for AN, however, set-shifting difficulties could be a possible endophenotype for this illness.

### Clinical implications

There has been an increased interest in incorporating findings from neuroscientific studies to the development of treatment strategies for AN. Cognitive Remediation Therapy (CRT) involves cognitive training which addresses difficulties with flexibility and global processing [33]. The aim of this treatment is to modify and improve these deficits, and apply new cognitive skills to eating-related tasks. A more recently developed treatment, Temperament-based treatment with support (TBT-S) [34] is also based on neuroscientific research. Here, the patients learn strategies to cope with their deficits, and learn how to use their personality traits in constructive ways. A complete understanding of the neuropsychological deficits in patients with AN, and whether they normalize with recovery, might influence how we develop such treatment strategies. An important issue is whether treatment should aim to alter these neuropsychological deficits, or whether the treatment should be adjusted to this specific neuropsychological thinking style.

### Limitations

Some weaknesses in the literature included in this review are important to consider. First, there is a lack of consensus in the eating disorder field on a standard definition of recovery of AN. This could offer challenges when comparing results across studies. In the current review, the majority of studies include a BMI > 18.5 and the absence of eating disorder symptoms as criteria for recovery. Although the majority of studies require that the recovered state should have been at least 1 year, other studies require a duration of 6 months and 3 months.

Conflicting results across studies could also be due to variations in clinical measures, such as duration of illness, severity degree and AN subtype (restrictive and binge/purge). Severely affected patients with a long duration of illness might have larger scarring effects compared to individuals with shorter duration of illness and less symptom severity. However, determining these variables accurately is difficult. Most studies do not define the term duration of illness and how it is assessed. In addition, duration of illness can be measured in various ways, such as by diagnosis date, what time the symptoms emerged, or what time the patient was entered into treatment. When it comes to severity degree, McGuire et al. [35] have proposed a theoretical model for the definition and conceptualization of severity of AN. According to this model, there should be a staging system, which is based on symptom severity. The main purpose of this system is to make appropriate treatment options, however it would also be beneficial for scientific purposes.

Few studies have separated patients into different subtypes. Neuropsychological difficulties could vary within the AN group, and by not separating individuals into subgroups, important findings could be overlooked. However, which group of patients that have neuropsychological difficulties is yet to be established, as studies are few and the findings are inconsistent. Lindner et al. [28] divided the AN-REC group by subtypes and found that the restricting group performed worse on a set-shifting task than HC. At the same time, Danner et al. [22] suggest that neuropsychological impairments are not a general problem for AN, but these are specific for a subpopulation within both ill and recovered patients. Therefore, there could be subgroups of patients that have problems with set-shifting which could be independent from AN diagnostic subtype, a topic in need of further investigation.

Other differences in study design, such as sample size, might also affect the results. Lack of power calculations estimating the sample size necessary to detect significant differences between groups, could contribute to conflicting findings. In the current review, studies within the central coherence domain showed various findings. One study that failed to find significant results had a sample size of fifteen in the AN-REC group. In addition, conflicting findings could also be reflected by the large variety of tests that have been used within the same cognitive domain.

An important area of research is focused upon which factors that distinguish adolescent patients from adults. Adolescents seem to have a better prognosis than adults [36]. Studies have consistently shown that adults with AN have altered executive functioning, and some of these functions might be trait related. However, when do state difficulties occur? Moreover, are they related to the maintenance of the illness? Future studies should compare neuropsychological performance in adolescents versus adults with AN. Future studies should also focus on differentiating predisposing factors and perpetuating factors. Predisposing factors include inherent characteristic, such as genetics and personality traits, while perpetuating factors are conditions that maintain the symptoms of the illness. Predisposing and perpetuating factors are not necessarily the same, and determining these could contribute to the development of better treatment strategies.

### Conclusion

The majority of studies in this review suggest that deficits in set-shifting persist after recovery, and could be trait related. Set-shifting difficulties could also be an endophenotype for AN as it is present in unaffected family members. Central coherence might also be trait related, however findings are inconsistent. Few studies have investigated decision-making in AN-REC, however existing studies suggest that decision-making is not impaired in AN-REC.

There is an urgent need for better treatment approaches for individuals with AN, and especially for adult patients. Novel treatment strategies based on neuroscience research are emerging, focusing on targeting the underlying mechanisms of the illness, including neuropsychological functioning. These new treatments include elements such as practicing and improving specific cognitive skills and teaching effective tools and strategies to manage eating disorder symptoms in a collaborative manner. The factors that sustain the illness are most likely different from the factors that contribute to the onset of the illness. It would therefore be beneficial to determining these factors so they could be correctly targeted in treatment.

## Data Availability

Not applicable.

## Abbreviations

AN: Anorexia nervosa
AN-REC: Anorexia nervosa recovered
BN: Bulimia nervosa
CRT: Cognitive remediation therapy
HC: Healthy controls
IGT: Iowa gambling task
RCFT: Rey complex figure test
TBT-S: Temperament-based treatment with support
TMT: Trail making test.
WCST: Wisconsin Card Sorting Test
WR-AN: Weight-recovered anorexia nervosa
